# Supplementary material for: Leptin induces TNFα-dependent inflammation in acquired generalized lipodystrophy and combined Crohn’s disease
Source: Nat Commun. 2019 Dec 10;10:5629. doi: 10.1038/s41467-019-13559-7 (PMC6904732; doi:10.1038/s41467-019-13559-7)
Supplement: Supplementary file 1 — Supplementary Information [file 41467_2019_13559_MOESM1_ESM.pdf]

Supplementary information

Leptin induces TNFα-dependent inflammation in acquired generalized lipodystrophy and combined Crohn's disease

Ziegler et. al.

Supplementary Note 1

A 21-year-old Caucasian male was repeatedly admitted to our hospital with acquired generalized lipodystrophy and fistulizing Crohn's disease (Montreal-classification: A1 L3 B2+B3p) during the period of 2016-2019. The patient had been diagnosed with acquired generalized lipodystrophy and an insulin resistant diabetes mellitus at the age of 4 years. The patient had subsequently developed acanthosis nigricans, as well as lipodystrophy-mediated hypertrophic cardiomyopathy and hepatopathy. Due to leptin deficiency, the patient had been intermittingly substituted with recombinant leptin[1] during the time periods of 2004-2005 and 2012-2016 and leptin substitution had been discontinued by the patient due to incompliance before admission to our hospital in 2016. At age of 11 (2006), the patient had been newly diagnosed with Crohn's disease and undergone hemicolectomy. Subsequently, he had received partial resection of the small intestine in 2007 before undergoing an ascendorectostomy in 2008. Due to the absence of fat storage in adipocytes, the patient had suffered from severe fatty liver degeneration, making allogenic liver transplantation necessary at age 15 in 2010, requiring a constant immune suppression with Mycophenolat-Mofetil and Tacrolimus ever since. At the initial admission to our department in 2016, a protective ileostomy was performed due to recurrent perianal abscess and fistulizing disease. In 3/2017, an enterocutaneous fistula at his right flank was diagnosed and MRI scans visualized the complete lack of visceral and subcutaneous adipose tissue (**Main Figure 1A**). After admission in 2017, the patient was restarted on daily injections with 2.5 mg recombinant *N*-methionylleptin (rLeptin, Myalept) to improve his insulin susceptibility and metabolic state before undergoing colectomy and combined terminal ileostomy due to aggravated Crohn's disease. At first endoscopy six months after surgery, the ileum was endoscopically as well as histologically inflamed. Due to the high fraction of TNFα producing cells under continuous leptin substitution, a treatment with TNFα antibodies (adalimumab) was initiated. Clinical and endoscopic remission was observed six months after initiating adalimumab therapy. The clinical history of the AGLCD patient is summarized in **Figure 1D** of the main text. None of the patient's relatives had a history of lipodystrophy or inflammatory bowel disease. A characterization of the patient's clinical immunologic state by measurement of various auto-antibodies, complement factors, immunoglobulin and immunoglobulin-subgroups revealed the presence of antinuclear auto-antibodies and rheumatoid factor as well as increased levels of Immunoglobulin A and G (**Supplementary Table 1**).

Supplementary Table 1. Levels of different auto-antibodies, immunoglobulins, immunoglobulin-subgroups and complement factors in the serum of the AGLCD patient were retrieved from his medical records and compiled from various time points. The reference range is indicated in brackets, altered values are highlighted in bold.

Parameter	AGLCD (normal range)
antinuclear antibodies (ANA)/Hep-2-IF	**1:620** (1:160)
c-ANCA	1.7 U/ml (<10.0 U/ml)
p-ANCA	2.0 U/ml (<5 U/ml)
anti-liver-kidney-microsome antibodies (LKM-1)	neg. (neg.)
anti-smooth muscle antibodies (ASMA)	neg. (neg.)
anti-mitochondrial antibodies (AMA)	neg. (neg.)
anti-transglutaminase antibodies (IgA)	2 .9 U/ml (<10.0 U/ml)
anti-transglutaminase antibodies (IgG)	3.9 U/ml (<10.0 U/ml)
anti-cardiolipin-antibodies (IgG)	**29.7** U/ml (<10 U/ml)
anti-cardiolipin-antibodies (IgM)	1.7 U/ml (<7 U/ml)
lupus anticoagulant	neg. (neg)
rheumatoid factor (IgA)	**42.7** U/ml (<20U/ml)
rheumatoid factor (IgG)	0 U/ml (<20U/ml)
C3-complement	930 mg/l (900-1800 mg/dl)
C4-complement	220 mg/l (100-400mg/l)
Immunoglobulin A	**13.2** g/l (0.7-4.0 g/l)
Immunoglobulin M	0.96 g/l (0.4-2.30g/l)
Immunoglobulin G	**26.7** g/l (7-16 g/l)
IgG1	**9.854** g/l (2.800-8.000 g/l)
IgG2	**8.094** g/l (1.120-5.700 g/l)
IgG3	0.897 g/l(0.240-1.250 g/l)
IgG4	0.013 g/l (0.052-1.250 g/l)
Alpha1 -proteinase-inhibitor	1.48 g/l (0.90-2.00 g/l)

Supplementary Table 2. **Mass cytometry antibody panels.**

For mass cytometry, two antibody panels containing 34 markers each were used. Where secondary antibodies were applied (against PE, FITC or rabbit; highlighted in bold), primary and secondary antibodies are listed both under the respective metal tag.

Panel A			Panel B		
**metal**	**target**	**clone / company**	**metal**	**target**	**clone / company**
^{142}Nd	CD19	HIB19 / Fluidigm	^{142}Nd	CD116	4H1 / Biolegend
^{143}Pr	HLA-DR	L243 / Fluidigm	^{143}Pr	HLA-DR	L243 / Fluidigm
^{144}Nd	CD38	HIT2 / Fluidigm	^{144}Nd	CD38	HIT2 / Fluidigm
^{145}Nd	CD4	RPA-T4 / Fluidigm	^{145}Nd	CD124	G077F6 / Biolegend
^{146}Nd	TNFα	Mab11 / Fluidigm	^{146}Nd	CD64	10.1 / Fluidigm
^{147}Sm	CD11c	Bu15 / Fluidigm	^{147}Sm	CD11c	Bu15 / Fluidigm
^{148}Nd	CD16 (FcγRIII)	3G8 / Fluidigm	^{148}Nd	CD16 (FcγRIII)	3G8 / Fluidigm
^{149}Sm	CCl2 (MCP-1)	5D3-F7 / Biolegend	^{149}Sm	CCl2 (MCP-1)	5D3-F7 / Biolegend
^{150}Nd	CD45	HI30 / Biolegend	^{150}Nd	CD45	HI30 / Biolegend
^{151}Eu	CD103	Ber-ACT8 / Fluidigm	^{151}Eu	CD68	Y1/82A / Biolegend
^{152}Sm	CD95 (Fas)	DX2 / Fluidigm	^{152}Sm	CD83	HB15e / Biolegend
^{153}Eu	CD62L	DREG-56 / Fluidigm	^{153}Eu	IL-6	MQ2-13A5 / Biolegend
^{154}Sm	CD3	UCHT1 / Fluidigm	^{154}Sm	CD3	UCHT1 / Fluidigm
^{155}Gd	CD56	B159 / Fluidigm	^{155}Gd	CD54	HA58 / Biolegend
^{156}Gd	CD195 (CCR5)	NP-6G4 / Fluidigm	^{156}Gd	CD274 (PD-L1)	29E.2A3 / Fluidigm
^{158}Gd	CD135	BV10A4H2 / Fluidigm	^{158}Gd	CD135	BV10A4H2 / Fluidigm
^{159}Tb	CD197 (CCR7)	G043H7 / Fluidigm	^{159}Tb	GM-CSF	BVD2-21C11 / Fluidigm
^{160}Gd	CD14	RMO52 / Fluidigm	^{160}Gd	CD163	GHI/61 / Biolegend
^{161}Dy	EMR1 (F4/80)	A10 / Bio-Rad	^{161}Dy	CD36	5-271/ Biolegend
^{162}Dy	CD8a	RPA-T8 / Fluidigm	^{162}Dy	FOXP3	PCH101 / Fluidigm
^{163}Dy	TGFβ	TW4-2F8 / Biolegend	^{163}Dy	TGFβ	TW4-2F8 / Biolegend
^{164}Dy	CD115	9-4D2-1E4 / Biolegend	^{164}Dy	Arginase-1	658922 / Fluidigm
^{165}Ho	T-bet (**PE**)	4B10 / eBioscience	^{166}Er	IL-10	JES3-9D7 / Fluidigm
	**PE**	PE001 / Fluidigm	^{167}Er	CD197 (CCR7)	G043H7 / Fluidigm
^{166}Er	IL-10	JES3-9D7 / Fluidigm	^{168}Er	CD206 (MMR)	15-2 / Fluidigm
^{167}Er	CD27	O323 / Fluidigm	^{169}Tm	CD33	WM53 / Fluidigm
^{168}Er	IFNγ	B27 / Fluidigm	^{170}Er	CD86	IT2.2 / Biolegend
^{169}Tm	CD33	WM53 / Fluidigm	^{171}Yb	CCR2	K036C2 / Biolegend
^{170}Er	CD86	IT2.2 / Biolegend	^{172}Yb	CX3CR1	2A9-1 / Fluidigm
^{171}Yb	CD192 (CCR2)	K036C2 / Biolegend	^{173}Yb	CD40	5C3 / Biolegend
^{172}Yb	CX3CR1	2A9-1 / Fluidigm	^{174}Yb	IL-8 (**FITC**)	E8N1 / Biolegend
^{173}Yb	CD40	5C3 / Biolegend		**FITC**	FIT22 /Fluidigm
^{174}Yb	PD-1	EH12.2H7 / Fluidigm	^{175}Lu	ADRP (**rabbit**)	polyclonal / Proteintech
^{176}Yb	IL-7R	A019D5 / Fluidigm		**rabbit**	polyclonal / Fluidigm
^{209}Bi	CD11b (Mac-1)	ICRF44 / Fluidigm	^{176}Yb	TREM2	237920 / R&D System
			^{209}Bi	CD11b (Mac-1)	ICRF44 / Fluidigm

Supplementary Table 3. **Flow cytometry antibodies.**

Fluorophore-labeled antibodies were used for various flow cytometry experiments and are listed by their human target in this table.

Fluorophore-labeled antibodies against human antigens			
**target**	**clone**	**fluorophore**	**company**
CD3	SK7	APC-eFluor780	eBioscience
CD3	HIT3a	PE	BioLegend
CD3	UCHT1	PerCP-Cy5.5	BioLegend
CD3	OKT3	APC	eBioscience
CD4	RPA-T4	APC	BD Biosciences
CD4	RPA-T4	Brilliant Violet 510	BioLegend
CD8a	RPA-T8	FITC	BioLegend
CD8a	SK1	PerCP- eFluor710	eBioscience
CD8a	RPA-T8	APC	eBioscience
CD11b	ICRF44	APC-Cy7	BioLegend
CD14	MφP9	APC	BD Biosciences
CD16	3G8	PE	BioLegend
CD25	M-A251	PerCP-Cy5.5	BD Biosciences
CD44	G44-26	PE-Cy7	BD Biosciences
CD56	TULY56	APC	eBioscience
CD56	TULY56	eFluor450	eBioscience
CD80	2D10.4	FITC	eBioscience
CD86	IT2.2	Brilliant Violet 421	BioLegend
CD137	4B4-1	PE	BioLegend
FOXP3	PCH101	PE	eBioscience
Granzyme B	GB11	Pacific Blue	BioLegend
IFNγ	4S.B3	APC-Cy7	BioLegend
IFNγ	4S.B3	FITC	BD Biosciences
Il-17A	BL168	Brilliant Violet 421	BioLegend
IL-17A	BL168	APC-Cy7	BioLegend
Perforin	delta G9	PE-Cy7	eBioscience
RORγt	AFKJS-9	APC	eBioscience
T-bet	4B10	PE-Cy7	eBioscience
TNFα	MAb11	PerCP-Cy5.5	BioLegend

Supplementary Table 4. **Histopathology antibodies.**

Antibodies used for histopathology on paraffin-embedded colonic tissue from the AGLCD patient and Crohn's disease patients are listed in this table.

**Histopathology antibodies against human antigens**		
**target**	**clone(s)**	**company**
CD45	2B11 + PD7/26	Dako
CD11b	EP1245Y	Abcam
CD206	5C11	LifeSpan BioSciences
TNFα	M1-C4	Sigma
iNOS	polyclonal rabbit	Invitrogen
CD86	polyclonal goat	R&D Systems
ADRP	polyclonal rabbit	Proteintech
CD163	10D6	Novocastra

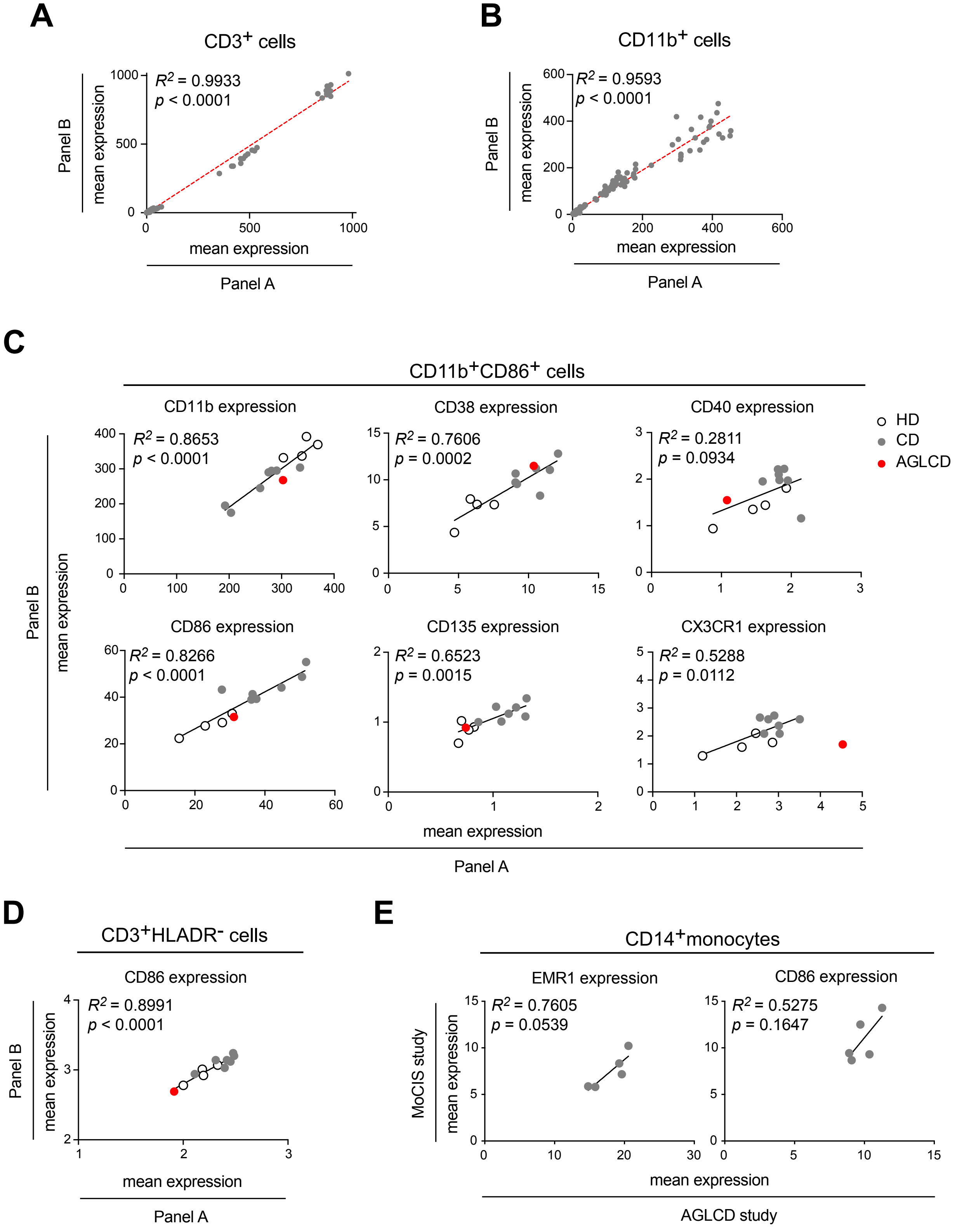
A
CD3+ cells
R2 = 0.9933
p < 0.0001
Panel B mean expression
mean expression Panel A
B
CD11b+ cells
R2 = 0.9593
p < 0.0001
Panel B mean expression
mean expression Panel A
C
CD11b+CD86+ cells
CD11b expression
R2 = 0.8653
p < 0.0001
CD38 expression
R2 = 0.7606
p = 0.0002
CD40 expression
R2 = 0.2811
p = 0.0934
HD
CD
AGLCD
CD86 expression
R2 = 0.8266
p < 0.0001
CD135 expression
R2 = 0.6523
p = 0.0015
CX3CR1 expression
R2 = 0.5288
p = 0.0112
Panel B mean expression
mean expression Panel A
D
CD3+HLADR- cells
CD86 expression
R2 = 0.8991
p < 0.0001
Panel B mean expression
mean expression Panel A
E
CD14+monocytes
EMR1 expression
R2 = 0.7605
p = 0.0539
CD86 expression
R2 = 0.5275
p = 0.1647
MoCIS study mean expression
mean expression AGLCD study

Supplementary Figure 1. Correlation of expression levels between markers stained in both mass cytometry panels.

Linear regression analysis comparing the levels of marker expression determined using different antibody panels within- and between-experiments. (**A**) Correlation of the mean expression of 16 markers on $CD3^+$ T cells of all samples (HD, CD and the AGLCD patients) investigated using antibody panel A and B. (**B**) Correlation of the mean expression of 16 markers on $CD11b^+$ cells of all samples (HD, CD and the AGLCD patients) determined using antibody panel A and B. (**C**) Correlation of the mean expression of CD11b, CD38, CD40, CD86, CD135 and CX3CR1 on $CD11b^+CD86^+$ cells of all samples (HD, CD and the AGLCD patients) determined using antibody panel A and B. (**D**) Correlation of the mean expression of CD86 on $CD3^+$HLA-DR^- T cells of all samples (HD, CD and the AGLCD patients) determined using antibody panel A and B. (**E**) Correlation of the mean expression of EMR1 and CD86 on $CD14^+$ monocytes of CD patients obtained from two different experiments (i.e. MoCIS study and AGLCD study). The MoCIS study was performed four months after the current AGLCD study. In the MoCIS study, immune phenotypes of $CD14^+$ monocytes of patients with early multiple sclerosis were compared with those of patients with Crohn's disease. Five of the CD patients analyzed in the MoCIS study were also analyzed in the AGLCD study. The healthy controls (HD) and antibody panels were however different between the two studies. The source data are provided as a Source Data file.

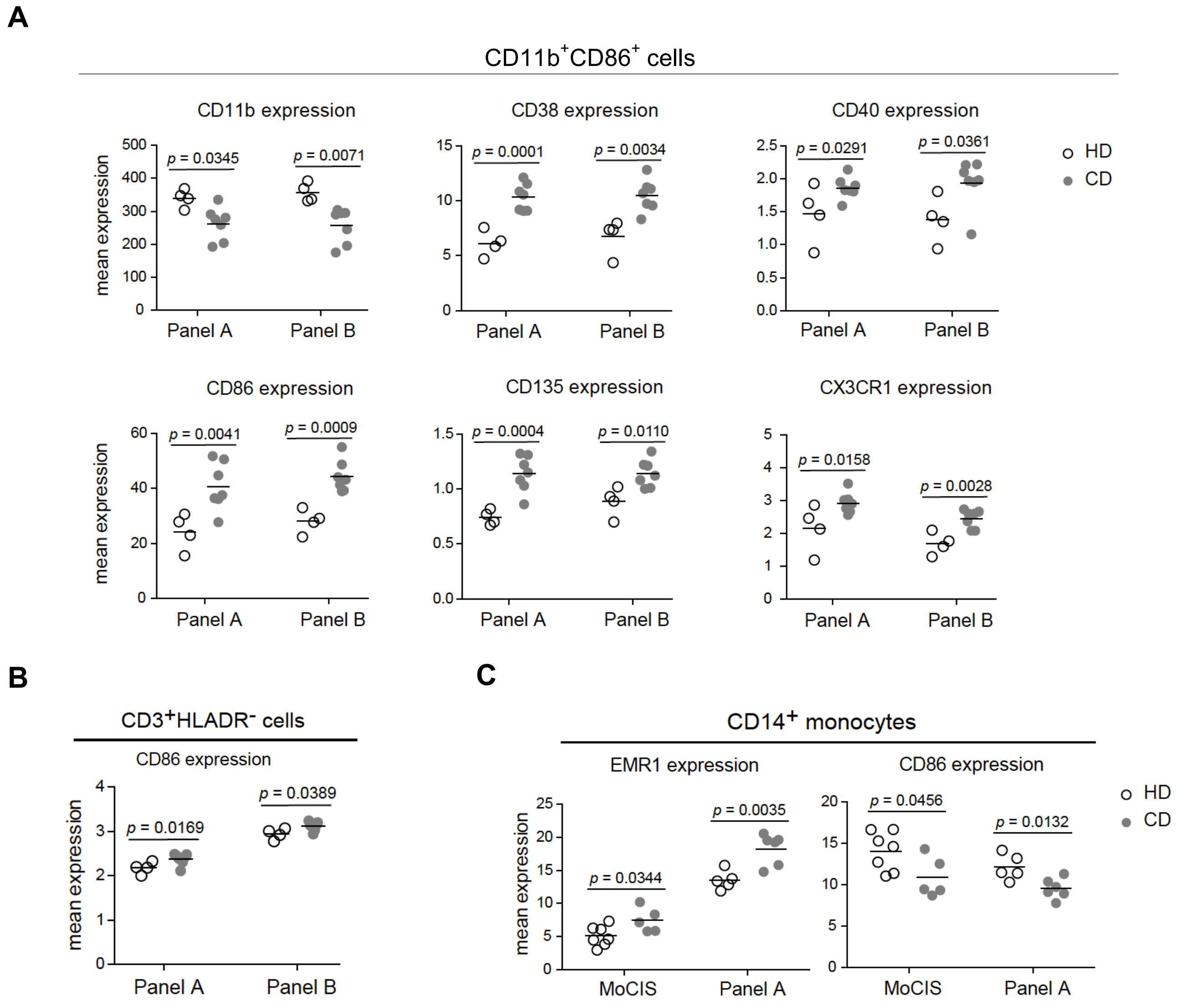

Supplementary Figure 2. Differences seen in mass cytometry are consistent between both panels and with different healthy controls.

The comparison of results of differential marker expressions determined using different antibody panels within- and between-experiments. (**A**) Differential expression of CD11b, CD38, CD40, CD86, CD135 and CX3CR1 on CD11b$^+$CD86$^+$ cells of patients with Crohn's disease (CD), determined using antibody panel A and B. (**B**) Differential expression of CD86 on CD3$^+$HLA-DR$^-$ cells of patients with Crohn's disease (CD), determined using antibody panel A and B. (**C**) Differential expression of EMR1 and CD86 on CD14$^+$ monocytes of patients with CD, determined in two different experiments (i.e. MoCIS study and AGLCD study), in which different antibody panels were applied. The source data are provided as a Source Data file.

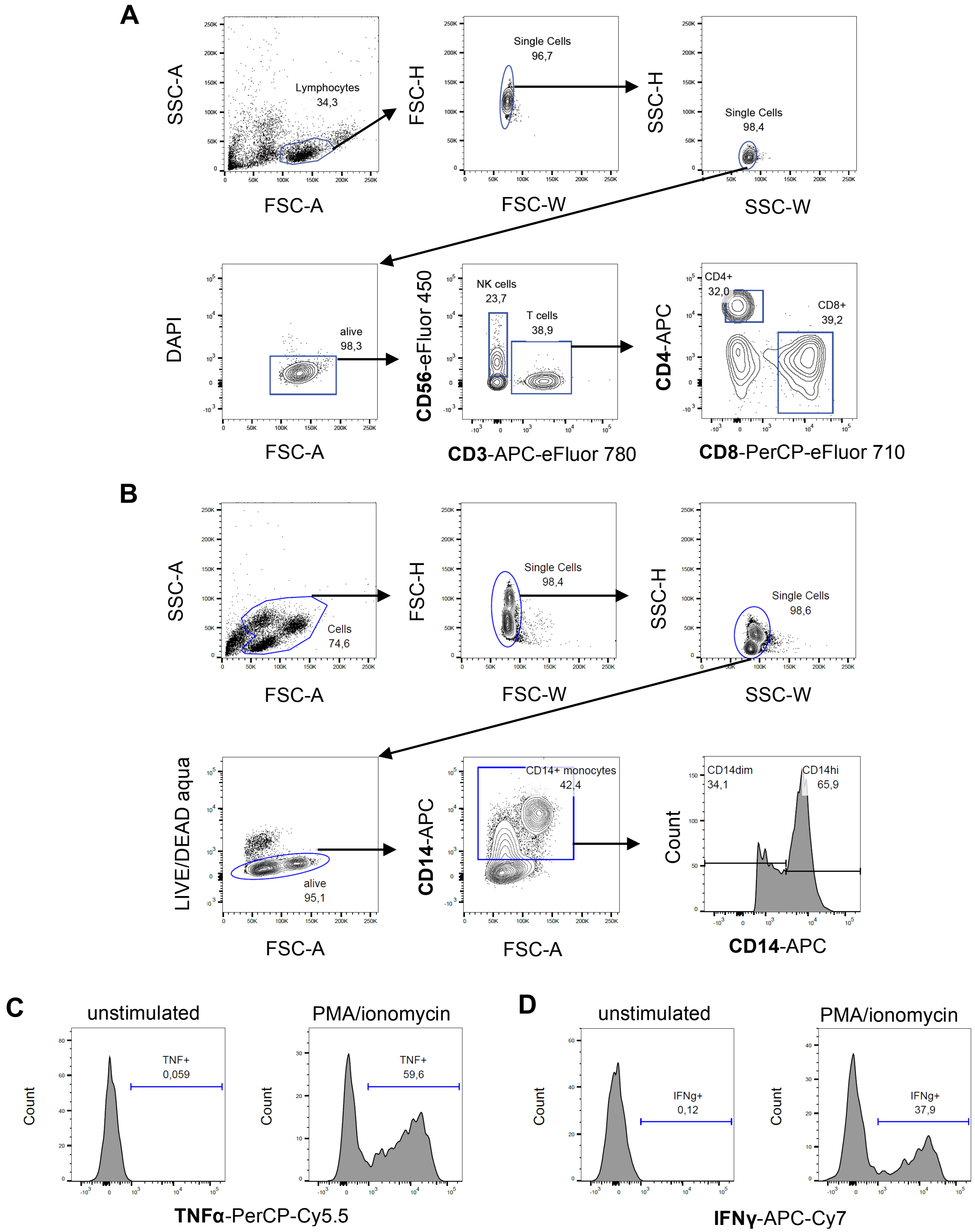

Supplementary Figure 3. Exemplary gating strategies for flow cytometry experiments.

Shown are exemplary gating strategies for flow cytometry to gate on (**A**) NK cells, $CD4^+$ and $CD8^+$ T cells as well as (**B**) $CD14^+$ monocytes. Exemplary gating strategies for cytokine production using unstimulated or PMA/ionomycin stimulated controls are shown for (**C**) TNFα and (**D**) IFNγ.

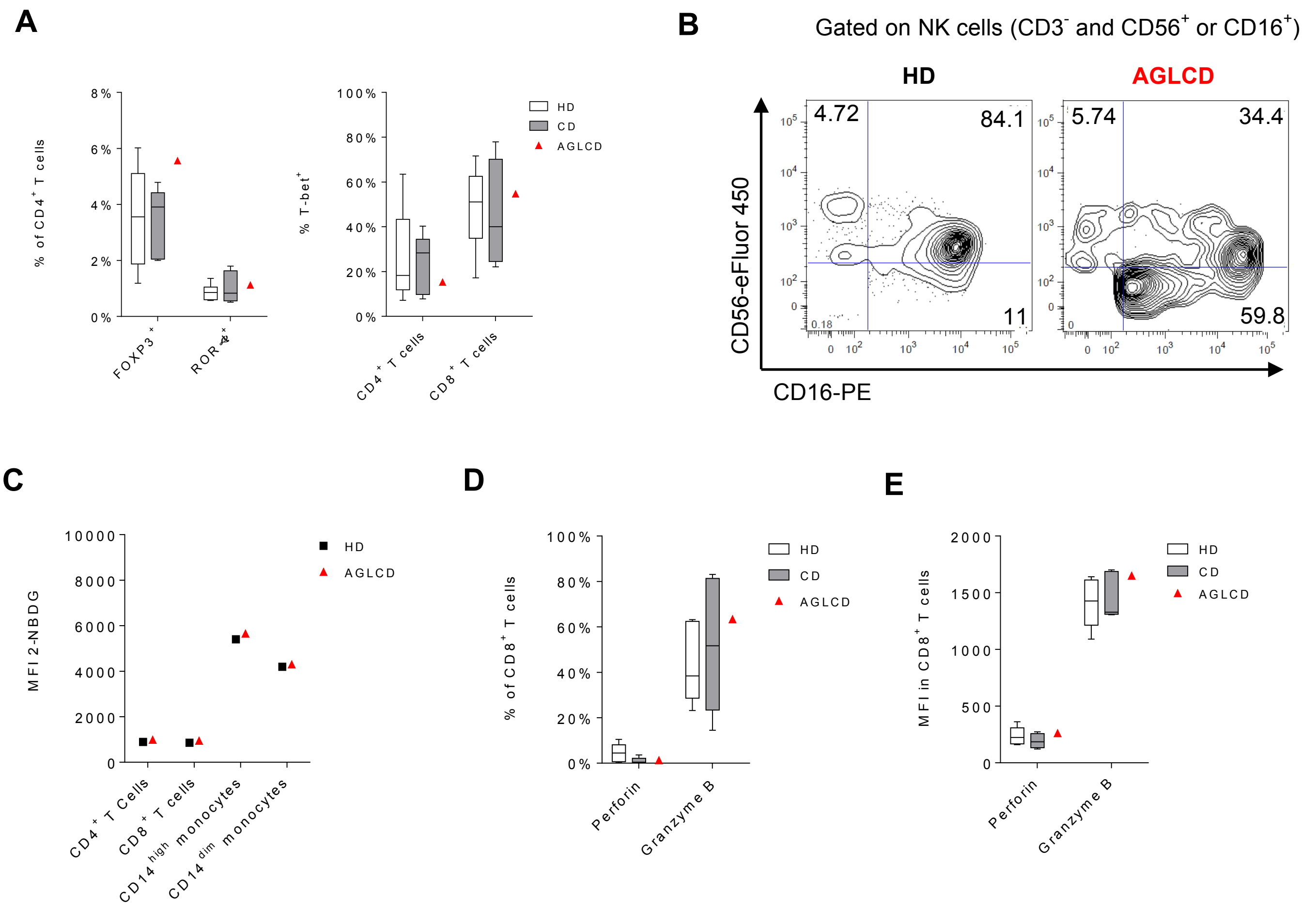

Supplementary Figure 4. Metabolic, functional and phenotypic assessment of peripheral blood mononuclear cells by flow cytometry*.*

(**A**) The percentages of T cells expressing distinct transcription factors was investigated in Crohn's disease patients (CD, n= 5) and healthy donors (HD, n=6). (**B**) Primary flow cytometry plots showing the expression pattern of CD56 and CD16 in NK cells ($CD3^-$ and $CD56^+$ or $CD16^+$ cells) in the AGLCD patient and a HD. (**C**) Difference in 2-NBDG mean fluorescence intensity (MFI) between stained sample and unstained control as a measure of glucose uptake in T cells subsets and monocytes from the AGLCD patient and HD (n=1). (**D**) Percentage of $CD8^+$ T cells producing the cytotoxic molecules perforin and granzyme B was measured and (**E**) the MFI for these molecules was calculated in CD (n=5) and HD (n=5). Whisker plots show median with min and max values. Two-tailed unpaired *t-tests* without correction for multiple comparison, considering P<0.05 as statistically significant. The source data are provided as a Source Data file.

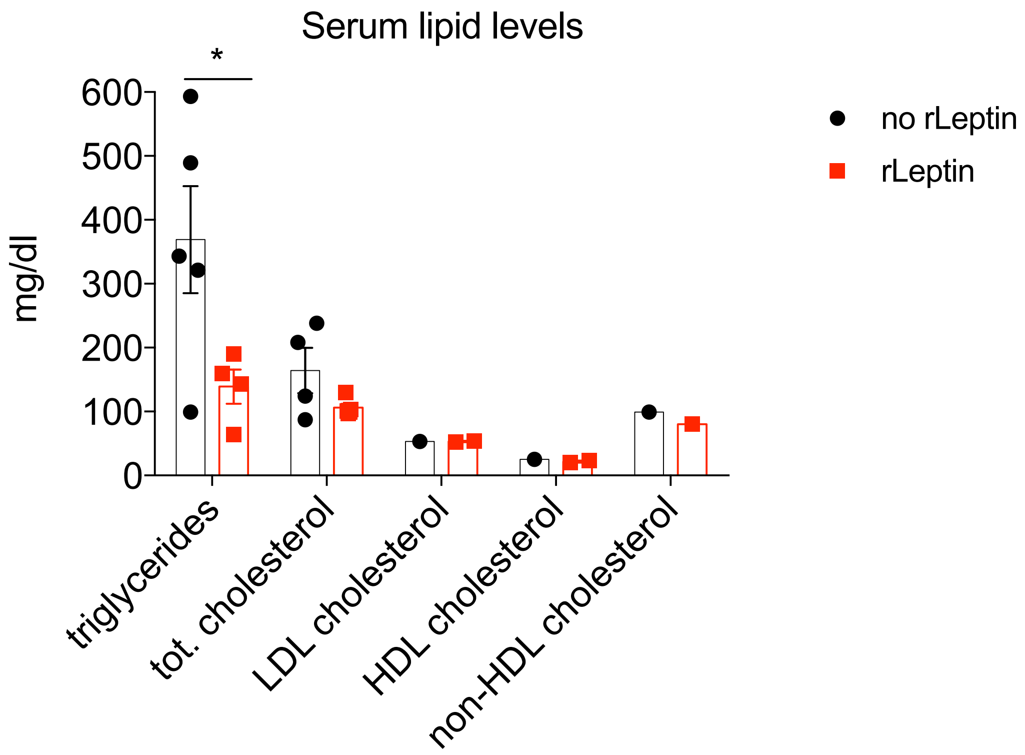

Supplementary Figure 5. Effects of rLeptin therapy on serum lipid levels.

Serum concentrations of lipids in the AGLCD patient in the presence or absence of rLeptin measured at various time points. Error bars show ±SEM, unpaired *t –test* with Welch's correction. * $p < 0.05$. The source data are provided as a Source Data file.

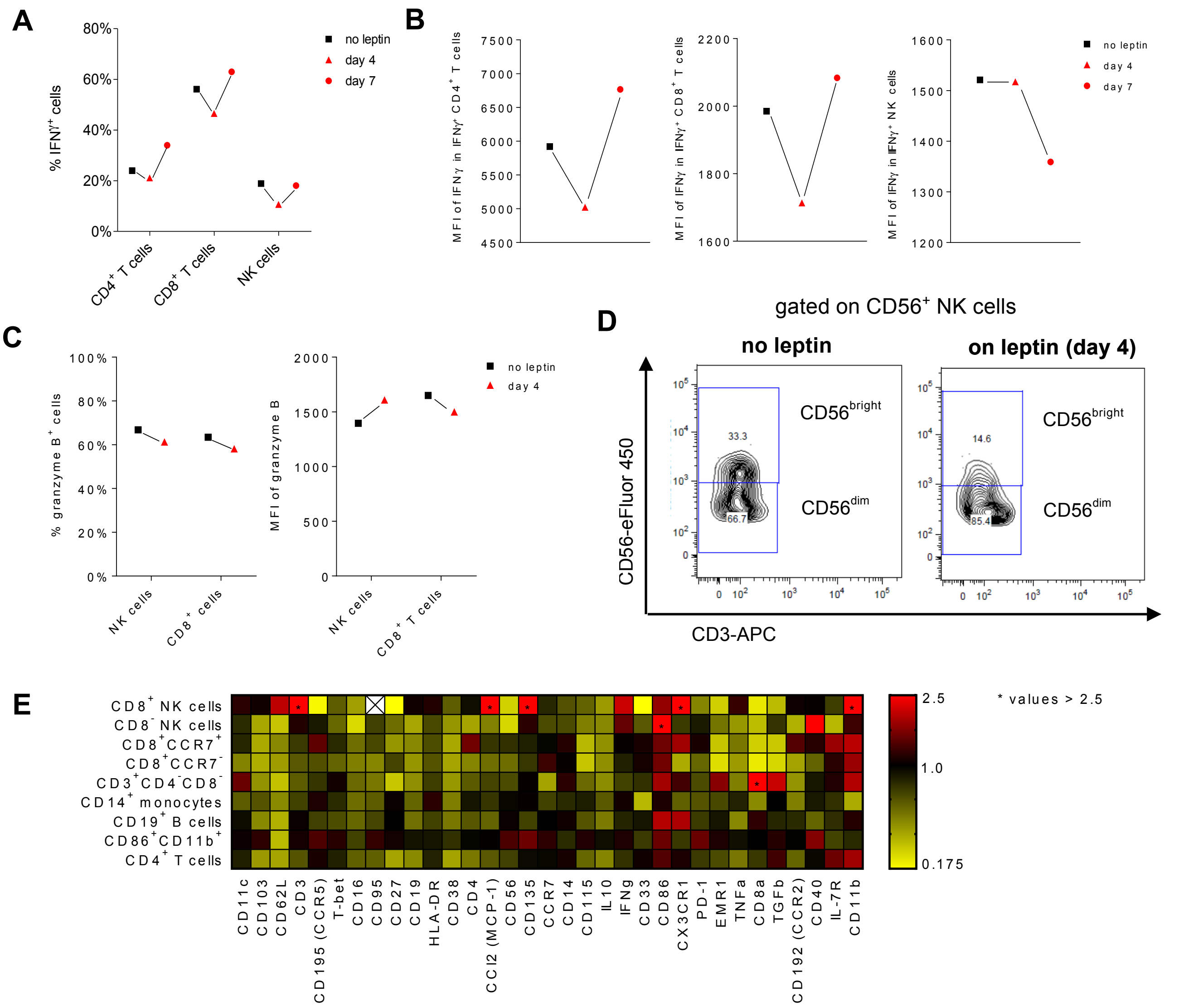

Supplementary Figure 6. **Effects of leptin therapy on peripheral blood mononuclear cells.** PBMCs were obtained from the AGLCD patient before and at different time points during substitution with rLeptin. PBMCs were analyzed by flow cytometry (**A-D**) and mass cytometry (**E**) for phenotypic and functional characteristics. (**A**) The percentage of cells positive for IFNγ and (**B**) the mean fluorescence intensity (MFI) of these cells were measured after *ex vivo* stimulation with PMA/ionomycin. (**C**) Perforin and granzyme B expression was assessed in NK and CD8$^+$ T cells by flow cytometry at day 4. (**D**) The frequencies of the two major NK cell subsets, CD56^{bright} and CD56^{dim}, were analyzed at day 4. (**E**) The expression of various markers was analyzed in different cell types by mass cytometry at day 7 and a heatmap of the fold-change following leptin therapy is shown, summarizing the results from antibody panel A. The heatmap from panel B is shown in **Figure 2** in the main text. The source data are provided as a Source Data file.

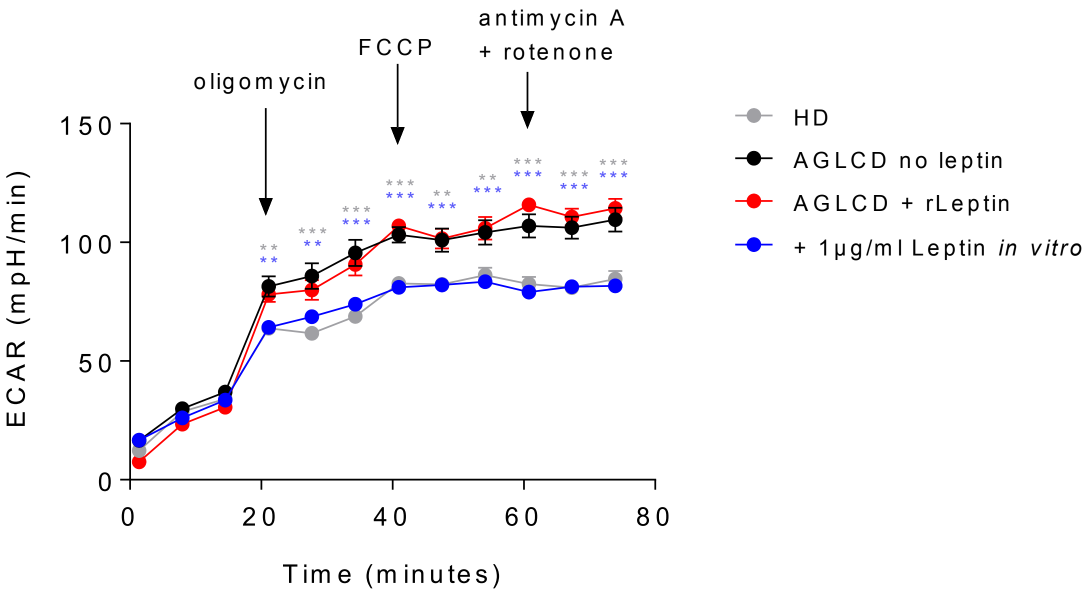

Supplementary Figure 7. Extracellular acidification rate of macrophages treated with serum from the AGLCD patient.

Extracellular acidification rate (ECAR) assessed by Seahorse analyses (Cell Mito Stress Kit) in monocyte-derived macrophages of a healthy donor differentiated in the presence of serum from the AGLCD patient before ("leptin-free") and after *in vitro* or *in vivo* leptin/rLeptin substitution, or in the presence of serum from a healthy donor (HD). ECAR data correspond to the oxygen consumption rates (OCR) reported in **Figure 3J** of the main text. Experiments were performed in at least triplicates, two-way ANOVA with post-tests and Holm-Sidak correction comparing against "AGLCD no leptin" as control were used for statistical analyses. Error bars represent ±SEM. * $p<0.05$, ** $p<0.01$, *** $p<0.001$. The source data are provided as a Source Data file.

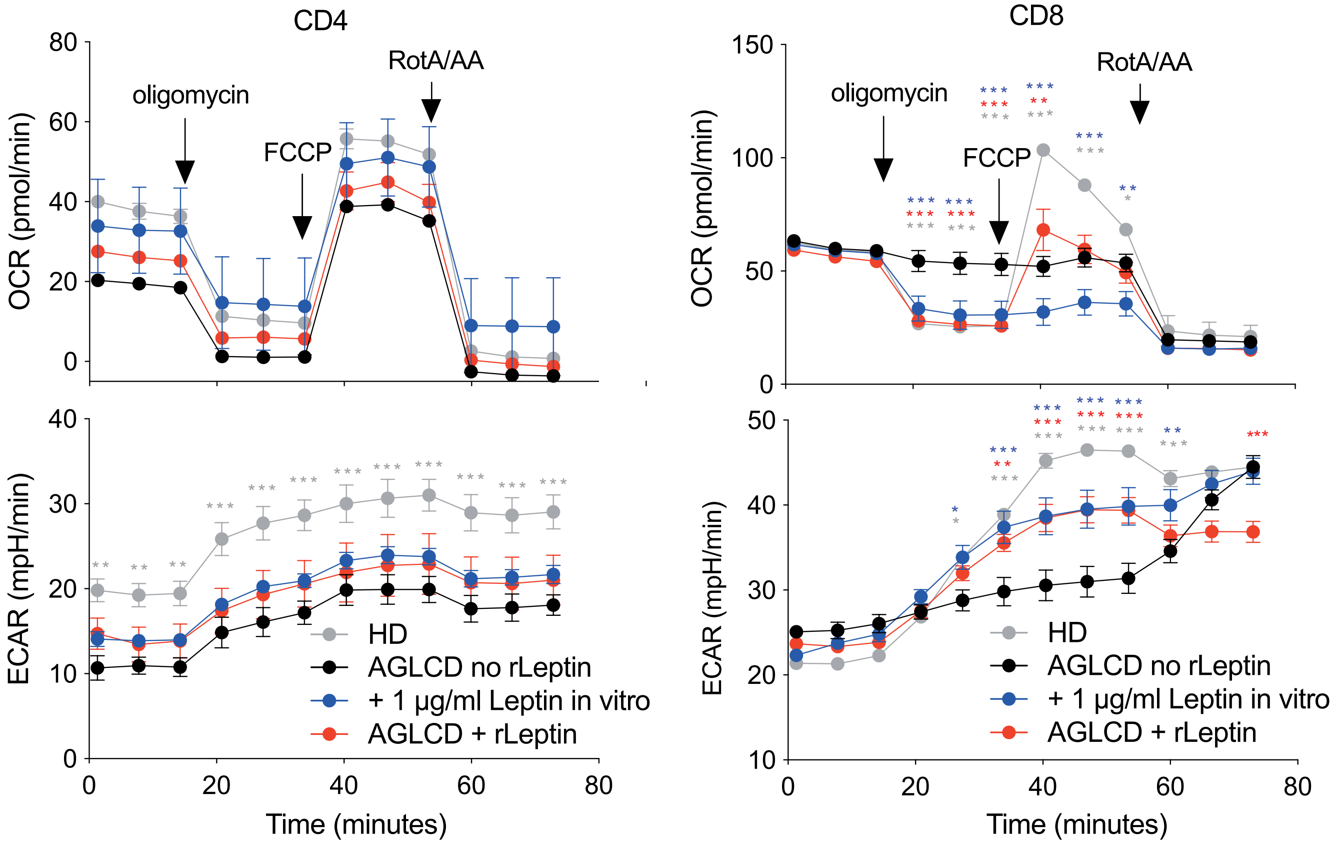

Supplementary Figure 8. Effects of rLeptin on the metabolism of T cells cultured in the presence of serum from the AGLCD patient.

$CD4^+$ and $CD8^+$ T cells were isolated from blood of healthy donors and were subsequently expanded by anti-CD3/anti-CD28 stimulation in the presence of serum from the AGLCD patient before ("leptin-free") and after *in vitro* or *in vivo* leptin/rLeptin substitution, or in the presence of serum from a healthy donor (HD). Oxygen consumption rate (OCR) and extracellular acidification rate (ECAR) were assessed by Seahorse analyses using the Cell Mito Stress Kit (Agilent). Experiments were performed in at least triplicates, statistical significance was assessed by two-way ANOVA with post-tests comparing against "AGLCD no leptin" as control with the Holm-Sidak correction for multiple comparison. Error bars showing ±SEM. * $p<0.05$, ** $p<0.01$, *** $p<0.001$. The source data are provided as a Source Data file.

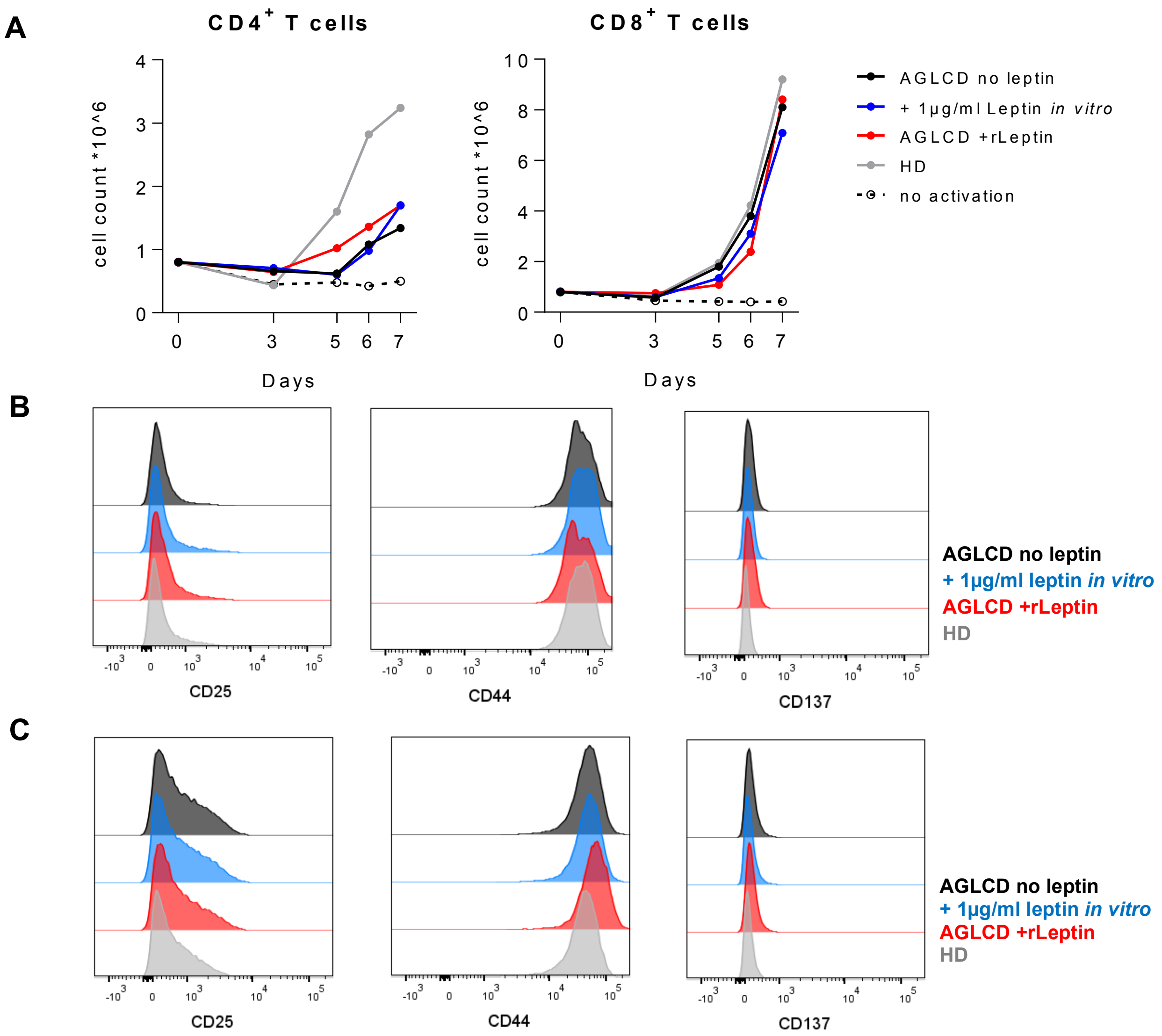

Supplementary Figure 9. Effects of rLeptin on proliferation and the activation of T cells cultured in the presence of serum from the AGLCD patient.

$CD4^+$ and $CD8^+$ T cells were isolated from blood of a healthy donor and were subsequently expanded by anti-CD3/anti-CD28 stimulation in the presence of serum from the AGLCD patient before ("leptin-free") and after *in vitro* or *in vivo* leptin/rLeptin substitution, or in the presence of serum from a healthy donor (HD). (**A**) Proliferation was assessed by counting viable cells. The expression of the activation markers CD25, CD44 and CD137 were assessed in technical duplicates in both (**B**) $CD4^+$ and (**C**) $CD8^+$ T cells, for which representative histoplots are shown. The source data are provided as a Source Data file.

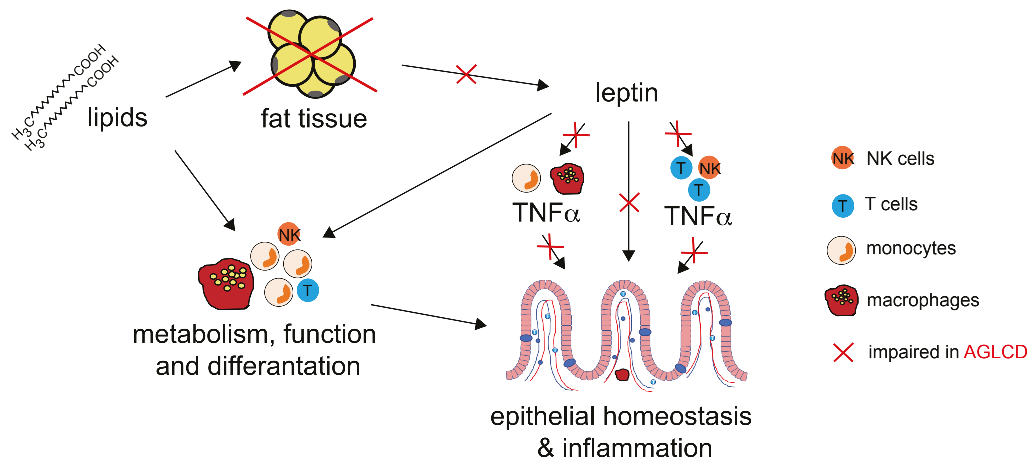

Supplementary Figure 10. Graphical summary of the effects of rLeptin substitution on the immune cell composition of the AGLCD patient.

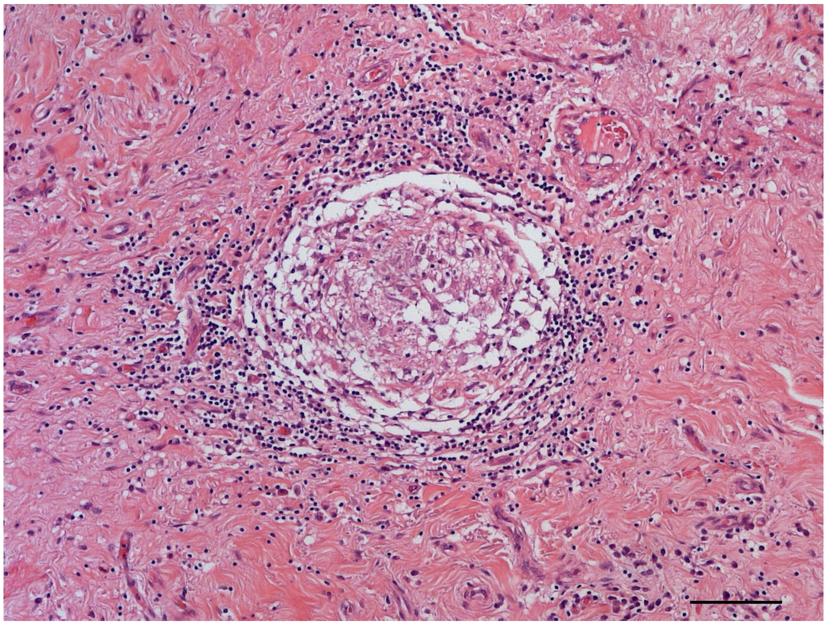

Supplementary Figure 11. Crohn's disease-typical granuloma in the submucosa of intestinal tissue from the AGLCD patient.

H&E staining of a tissue sample derived from the resected colon of the AGLCD patient showing submucosal granuloma formation, which is a typical feature of active Crohn's disease. Please also note the total absence of adipocytes in the specimen. The indicated scale bar depicts 200 µm.

Supplementary References

1. Oral, E.A., Simha, V., Ruiz, E., Andewelt, A., Premkumar, A., Snell, P., Wagner, A.J., DePaoli, A.M., Reitman, M.L., Taylor, S.I., et al. (2002). Leptin-replacement therapy for lipodystrophy. N Engl J Med *346*, 570-578.
