# Supplementary material for: Leptin induces TNFα-dependent inflammation in acquired generalized lipodystrophy and combined Crohn’s disease
Source: Nat Commun. 2019 Dec 10;10:5629. doi: 10.1038/s41467-019-13559-7 (PMC6904732; doi:10.1038/s41467-019-13559-7)
Supplement: Supplementary file 3 — Reporting Summary [file 41467_2019_13559_MOESM3_ESM.pdf]

nature research

Corresponding author(s): Britta Siegmund, Carl Weidinger

Last updated by author(s): Oct 22, 2019

Reporting Summary

Nature Research wishes to improve the reproducibility of the work that we publish. This form provides structure for consistency and transparency in reporting. For further information on Nature Research policies, see Authors & Referees and the Editorial Policy Checklist .

Statistics

For all statistical analyses, confirm that the following items are present in the figure legend, table legend, main text, or Methods section.

n/a	Confirmed	
☐	☒	The exact sample size (*n*) for each experimental group/condition, given as a discrete number and unit of measurement
☐	☒	A statement on whether measurements were taken from distinct samples or whether the same sample was measured repeatedly
☐	☒	The statistical test(s) used AND whether they are one- or two-sided<br>*Only common tests should be described solely by name; describe more complex techniques in the Methods section.*
☐	☒	A description of all covariates tested
☐	☒	A description of any assumptions or corrections, such as tests of normality and adjustment for multiple comparisons
☐	☒	A full description of the statistical parameters including central tendency (e.g. means) or other basic estimates (e.g. regression coefficient) AND variation (e.g. standard deviation) or associated estimates of uncertainty (e.g. confidence intervals)
☐	☒	For null hypothesis testing, the test statistic (e.g. *F*, *t*, *r*) with confidence intervals, effect sizes, degrees of freedom and *P* value noted<br>*Give P values as exact values whenever suitable.*
☒	☐	For Bayesian analysis, information on the choice of priors and Markov chain Monte Carlo settings
☒	☐	For hierarchical and complex designs, identification of the appropriate level for tests and full reporting of outcomes
☒	☐	Estimates of effect sizes (e.g. Cohen's *d*, Pearson's *r*), indicating how they were calculated

Our web collection on statistics for biologists contains articles on many of the points above.

Software and code

Policy information about availability of computer code

Data collection	The CyTOF acquisition software 6.5.236 and the flow cytometry acquisition BD FACSDiva Software 6.1.3 were used for cytometric data collection. Multispectral images were acquired using a Vectra® 3 imaging system (Perkin Elmer). Seahorse data was acquired on a Seahorse XFe96 Analyzer equipped with the Wave software version 2.4.1.1 (Agilent).
Data analysis	Statistical analysis and graphic data representation were done using GraphPad Prism version 7.00. Exome-sequencing was analyzed using the Alissa Interpret software Agilent). Mass and Flow Cytometry data were analyzed using the Cytobank (www.cytobank.org) or the FlowJo software package (V10.1). Scratch assays were analyzed using the MiToBo Scratch Assay Analyzer (ImageJ). BCA data were analyzed using the FCAP Array™ software V3.0 (BD Biosciences).

For manuscripts utilizing custom algorithms or software that are central to the research but not yet described in published literature, software must be made available to editors/reviewers. We strongly encourage code deposition in a community repository (e.g. GitHub). See the Nature Research guidelines for submitting code & software for further information.

Data

Policy information about availability of data

All manuscripts must include a data availability statement. This statement should provide the following information, where applicable:

- Accession codes, unique identifiers, or web links for publicly available datasets
- A list of figures that have associated raw data
- A description of any restrictions on data availability

All mass and flow cytoemtric data sets as well as exom sequencing results generated and analyzed during the current study are available from the corresponding authors on reasonable request that does not include confidential patient information.

Field-specific reporting

Please select the one below that is the best fit for your research. If you are not sure, read the appropriate sections before making your selection.

- [x] Life sciences
- [ ] Behavioural & social sciences
- [ ] Ecological, evolutionary & environmental sciences

For a reference copy of the document with all sections, see nature.com/documents/nr-reporting-summary-flat.pdf

Life sciences study design

All studies must disclose on these points even when the disclosure is negative.

Sample size	No priori statistical methods were used to predetermine sample sizes due to sample accessibility and insufficient previous data to enable this.
Data exclusions	One patient was excluded from all analyses, after ulcerative colitis had been histologically diagnosed instead of Crohn's disease. No other data were excluded.
Replication	The expression of lymphocyte and monocyte key markers of each individual sample were measured twice (antibody panel A and B) and were reliably reproduced as specified in Supplementary Figure 1 and 2. To demonstrate the robustness and degree of reproducibility of our mass cytometric findings and to exclude batch effects, we not only controlled for within-day variations by comparing 16 overlapping immune markers included in both our CyTOF antibody panels but also controlled for day-to-day variations by comparing the results of our current study to data obtained from an independent study. The results are shown in Supplementary Figures 1 and 2.
Randomization	No method of randomization was used in this study.
Blinding	No blinding was done for CYTOF analyses, since we performed unsupervised data processing and data analysis, thus excluding the possibility of biased results. For immunohistochemistry, all evaluations were performed in a blinded manner.

Reporting for specific materials, systems and methods

We require information from authors about some types of materials, experimental systems and methods used in many studies. Here, indicate whether each material, system or method listed is relevant to your study. If you are not sure if a list item applies to your research, read the appropriate section before selecting a response.

Materials & experimental systems

n/a	Involved in the study	
[ ]	[x]	Antibodies
[ ]	[x]	Eukaryotic cell lines
[x]	[ ]	Palaeontology
[x]	[ ]	Animals and other organisms
[ ]	[x]	Human research participants
[ ]	[x]	Clinical data

Methods

n/a	Involved in the study	
[x]	[ ]	ChIP-seq
[ ]	[x]	Flow cytometry
[x]	[ ]	MRI-based neuroimaging

Antibodies

Antibodies used

All antibodies including clone name and supplier name are described within the Supplementary information.

Validation

All antibodies used for mass and flow cytometry have previously been validated in our group in human leukocytes (Böttcher C et al., Nature Neuroscience 2019, Kredel LI et al., Journal of Crohns and Colitis, 2019) . All immunohistochemistry antibodies were pre-tested by the manufacturer and have been used in other publications (see the websites of the manufacturers for further information). All antibodies were titrated in our group for optimal dilution.

Immunohistochemistry antibodies:
CD45 (1:5000 / 2B11+PD7/26 / Dako); CD11b (1:5000 / EP1245Y / Abcam); CD206 (1:2000 / 5C11 / LifeSpan Biosciences); TNFa (1:5000 / M1-C4 / Sigma-Aldrich); iNOS (1:1000 / Polyclonal / Invitrogen); CD86 (1:1000 / Polyclonal / R&D System); ADRP (1:2000 / Polyclonal / Proteintech); CD163 (1:3000 / 10D6 / Novocastra)

FACS antibodies:
CD3 (1:100, SK7 (APC-eFluor780, eBioscience); HIT3a (PE, Biolegend); UCHT1 (PerCP-Cy5.5, Biolegend) or 1:200, OKT3 (APC, eBioscience)); CD4 (1:40, RPA-T4 (APC, BD Biosciences or Brillant Violet 510, Biolegend)); CD8a (1:20, RPA-T4 (FITC, Biolegend or APC, eBioscience); 1:100, SK1 (PerCP-eFluor710, eBioscience)); CD11b (1:100, ICRF44 (APC-Cy7, Biolegend)); CD14 (1:40, MpP9 (APC, BD Bioscience)); CD16 (1:40, 3G8 (PE, Biolegend)); CD25 (1:50, M-A251 (PerCP-Cy5.5, BD Biosciences)); CD44 (1:100, G44-26 (PE-Cy7, BD Biosciences)); CD56 (1:200, TULY56 (APC or eFluor450, eBioscience)); CD80 (1:40, 2D10.4 (FITC, eBioscience)); CD86 (1:100, IT2.2 (Brillant Violet 421, Biolegend)); CD137 (1:40, 4B4-1 (PE, Biolegend)); FOXP3 (1:40, PCH101 (PE,

eBioscience)); Granzyme B (1:500, GB11 (Pacific Blue, Biolegend)); IFN-g (1:40, 4S.B3 (APC-Cy7, Biolegend or FITC, BD Biosciences)); IL-17A (1:50, BL168 (Brillant Violet 421 or APC-Cy7, Biolegend)); Perforin (1:100, delta G9 (PE-Cy7, eBioscience)); RORgt (1:100, AFKJS-9 (APC, eBioscience)); T-bet (1:100, 4B10 (PE-Cy7, eBioscience)); TNF-a (1:50, MAb11 (PerCP-Cy5.5))

CyTOF antibodies:
CD45 (1:100, HI30 / Cat#304045 / Biolegend); CD19 (1:100, HIB19 / Cat#: 3142001B / Fluidigm); HLA-DR (1:100, L243 / Cat#: 3143013B / Fluidigm); CD11b (1:100, ICRF44 / Cat#: 3209003B / Fluidigm); CD64 (1:100, 10.1 / Cat#: 3146006B / Fluidigm); CD11c (1:100, Bu15 / Cat#: 3147008B / Fluidigm); CD16 (1:100, 3G8 / Cat#: 3148004B / Fluidigm); CCl2 (1:200, 5D3-F7 / Cat#: 502601 / Biolegend); CD68 (1:100, Y1/82A / Cat#: 333801 / Biolegend); TNF-a (1:100, Mab11 / Cat#: 3146010B / Fluidigm); CD3 (1:100, UCHT1 / Cat#: 3154003B / Fluidigm); CD56 (1:100, B159 / Cat#: 3155008B / Fluidigm); CCR5 (1:100, NP-6G4 / Cat#: 3156015A / Fluidigm); CD163 (1:100, GHI/61 / Cat#: 333602 / Biolegend); EMR1 (F4/80, A10 / Cat#: MCA2674GA / Bio-Rad); TGF-b(1:100, TW4-2F8 / Cat#: 349602 / Biolegend); CD115 (1:100, 9-4D2-1E4 / Cat#: 347302 / Biolegend); T-bet-PE (1:100, 4B10 / Cat#: 12-5825-82 / eBioscience); PE (1:100, PE001 / Cat#: 3165015B / Fluidigm); IL-10 (1:100, JES3-9D7 / Cat#: 3166008B / Fluidigm); CD206 (1:100, 15-2 / Cat#: 3168008B / Fluidigm); CD33 (1:100, WM53 / Cat#: 3169010B / Fluidigm); CD86 (1:100, IT2.2 / Cat#: 305402 / Biolegend); CCR2 (1:100, K036C2 / Cat#: 357202 / Biolegend); CX3CR1 (1:100, 2A9-1 / Cat#: 3172017B / Fluidigm); CD14 (1:100, RMO52 / Cat#: 3160006B / Fluidigm); TREM2 (1:100, 237920 / Cat#: MAB17291-100 / R&D Systems); CD116 (1:100, 4HI / Cat#: 305902 / Biolegend); IL-6 (1:100, MQ2-13A5 / Cat#: 501101 / Biolegend); CD54 (ICAM1) (1:100, HA58 / Cat#: 353102 / Biolegend); PD-L1 (1:100, 29E.2A3 / Cat#: 3156026B / Fluidigm); GM-CSF (1:100, BVD2-21C11 / Cat#: 3159008B / Fluidigm); CCR7 (1:100, G043H7 / Cat#: 3159003A and 3167009A / Fluidigm); CD4 (1:100, RPA-T4 / Cat#: 3145001B / Fluidigm); PD-1 (1:100, EH12.2H7 / Cat#: 3174020B / Fluidigm); CD95 (1:100, DX2 / Cat#: 3152017B / Fluidigm); CD127 (1:100, A019D5 /Cat#: 3176004B / Fluidigm); FITC (1:100, FIT22 / Cat#: 3174006B / Fluidigm); Rabbit (1:100, Polyclonal / Cat#: 3175002G / Fluidigm); CD38 (1:100, HIT2 / Cat#: 3144014B / Fluidigm); CD124 (1:100, G077F6 / Cat#: 355002 / Biolegend); CD103 (1:100, Ber-ACT8 / Cat#: 3151011B / Fluidigm); CD62L (1:100, DREG-56 / Cat#: 3153004B / Fluidigm); CD83 (1:100, HB15 / Cat#: 305302 / Biolegend); CD135 (1:100, BV10A4H2 / cat#: 3158019B / Fluidigm); CD8a (1:100, RPA-T8 / Cat#: 3162015B / Fluidigm); FOXP3 (1:100, PCH101 / Cat#: 3162011A / Fluidigm); Arginase I (1:100, 658922 / Cat#: 3164012B / Fluidigm); CD36 (1:100, 5-271 / Cat#: 336202 / Biolegend); CD27 (1:100, O323 / Cat#: 3167002B / Fluidigm); IFNg (1:100, B27 / Cat#: 3168005B / Fluidigm); CD40 (1:100, 5C3 / Cat#: 334302 / Biolegend); IL-8-FITC (1:100, E8N1 / Cat#: 511406 / Biolegend)

Eukaryotic cell lines

Policy information about cell lines

Cell line source(s)	The T84 cell line (ATCC CCL-248) was obtained from ATCC.
Authentication	The cell line was not authenticated.
Mycoplasma contamination	The cell line tested negative for mycoplasma contamination.
Commonly misidentified lines (See ICLAC register)	No commonly misidentified cell line was used.

Human research participants

Policy information about studies involving human research participants

Population characteristics

For isolation of peripheral blood mononuclear cells and serum ELISA, blood was collected from patients with Crohn's disease who were 27-63 years old. All patients had clinically active Crohn's disease at the time of collection and none of the patients received anti-TNFa therapy at the time of blood collection.

For a detailed medical history of the AGLCD patient, please see the Case Report in the Supplementary Information.

Healthy donors were 24-47 years old and had no self-reported medical conditions at the time of blood collection.

For immunohistochemistry, paraffin-embedded intestinal tissue of patients with confirmed Crohn's disease was provided by the Central Biomaterial Bank (ZeBanC) of the Charité and compared to resected intestinal tissue of the AGLCD patient.

Recruitment

Blood samples from patients and healthy donors were obtained at the Dept. of Gastroenterology, Infectiology and Rheumatology, CBF, Charité - Universitätsmedizin Berlin. Parraffin-embedded intestinal tissue was provided by the Central Biomaterial Bank (ZeBanC) of the Charité and the Berlin Institute of Health. Permission to use blood and tissue samples as well as medical records were received from the Ethical Committee of the Charité, Berlin

Ethics oversight

The study was approved by the local ethics commitee of the Charité, Berlin.

Note that full information on the approval of the study protocol must also be provided in the manuscript.

Clinical data

Policy information about clinical studies

All manuscripts should comply with the ICMJE guidelines for publication of clinical research and a completed CONSORT checklist must be included with all submissions.

Clinical trial registration	n/a
Study protocol	n/a
Data collection	Blood samples were collected at the Dept. of Gastroenterology, Infectiology and Rheumatology, CBF, Charité - Universitätsmedizin Berlin between April, 2017 and June, 2019. Clinical data of the patients were retrieved from their medical records.
Outcomes	No primary or secondary outcome measures were pre-defined.

Flow Cytometry

Plots

Confirm that:

- [x] The axis labels state the marker and fluorochrome used (e.g. CD4-FITC).
- [x] The axis scales are clearly visible. Include numbers along axes only for bottom left plot of group (a 'group' is an analysis of identical markers).
- [x] All plots are contour plots with outliers or pseudocolor plots.
- [x] A numerical value for number of cells or percentage (with statistics) is provided.

Methodology

Sample preparation	Please see Materials and Methods for details (p.15)
Instrument	Please see Materials and Methods for details (p. 17)
Software	Please see Materials and Methods for details (p. 17)
Cell population abundance	No cell sorting was performed for this study.
Gating strategy	Please see Materials and Methods for a description of gating strategies for mass and flow cytometry (p.24). A Supplementary Figure exemplifying gating strategies is included as Supplementary Figure 3.

- [x] Tick this box to confirm that a figure exemplifying the gating strategy is provided in the Supplementary Information.
